# Effects of Perfluorooctane Sulfonic Acid Exposure on Intestinal Microbial Community, Lipid Metabolism, and Liver Lesions in Mice

**DOI:** 10.3390/ijms26062648

**Published:** 2025-03-14

**Authors:** Qianfeng Chen, Yulang Chi, Qingyu Zhu, Nana Ma, Lingli Min, Shouping Ji

**Affiliations:** 1 College of Oceanology and Food Science, Quanzhou Normal University, Quanzhou 362000, China; 2 College of Life Science, Hebei University, Baoding 071002, China; 3 College of Resources and Environmental Science, Quanzhou Normal University, Quanzhou 362000, China

**Keywords:** PFOS, intestinal microbiota, physiological health, dyslipidemia, liver lesions

## Abstract

Perfluorooctane sulfonic acid (PFOS) is a persistent organic pollutant that has attracted much attention due to its wide environmental distribution and potential toxicity. Intestinal microbiota is an important regulator of host health, and its composition and metabolic function are easily interfered with by environmental pollutants. In this study, the effects of PFOS exposure on gut microbiota, lipid metabolism, and host health were investigated in mice. The results showed that PFOS exposure did not significantly change α diversity, but significantly affected the β diversity and community structure of intestinal microflora in mice. At the taxonomic level, the ratio of Firmicutes to Bacteroidetes decreased, and the changes in the abundance of specific bacteria were closely related to liver diseases and lipid metabolism disorders. PFOS exposure also interfered with the gut–liver axis mechanism, increased blood lipids and liver function related indicators in mice, and induced intestinal and liver histological lesions. This study revealed the toxic mechanism of PFOS mediated by intestinal microbiota, providing a new research perspective for health problems caused by environmental pollutants and theoretical support for the formulation of relevant public health policies.

## 1. Introduction

Perfluoroalkyl and polyfluoroalkyl substances (PFASs) are a diverse group of synthetic chemicals that have been widely used in industrial applications and consumer product production for over 80 years due to their oil, water, and heat resistance, including for chemical, electronics, photographic, military, aviation, agricultural, packaging, and textile applications [1,2]. While there are thousands of different PFAS, only a few are monitored and subject to regulation, and perfluorooctane sulfonic acid (PFOS) is one of them [3]. As a common surfactant and plasticizer, PFOS is frequently used in industry, which has also contributed to serious environmental pollution in recent years [4]. Due to its extremely persistent nature, PFOS is the most difficult organic pollutant to degrade and can remain in the environment for a long time. At the same time, PFOS can accumulate in organic organisms. There is considerable evidence that organisms in the aquatic food chain have a strong PFOS accumulation effect [5]. PFOS in water is transferred to higher organisms, including humans, through the accumulation effect in aquatic organisms and food chains [6].

Epidemiological studies have shown that long-term exposure to certain environmental chemicals may lead to the development of metabolic disorders, which may be associated with disruption of gut microbial community structure and function [7,8]. Previous studies have shown that a certain amount of PFOS affects the gut microbiota and disrupts the gut environment by disrupting the gut barrier in mice [9]. A growing body of research suggests that disruption of the gut microbiota is closely associated with the onset and development of inflammatory bowel disease (IBD). Patients with IBD have abnormal gut microbiota composition compared to healthy individuals, particularly in terms of the abundance and diversity of the microbiota [10]. Numerous studies have also shown that IBD patients are often accompanied by intestinal microbiota disturbances, with a decrease in Firmicutes and an increase in Bacteroides [11,12]. Faced with the challenge of diagnosing non-alcoholic fatty liver disease (NAFLD) due to a lack of non-invasive methods, it remains uncertain whether PFOS causes liver steatosis in humans. In adult rodents and monkeys, PFOS administration has been shown to reduce body weight, decrease serum total cholesterol, and lead to hepatocytes hypertrophy and lipid cavitation [13,14,15]. Furthermore, gut microbiota is highly correlated with liver function, and the so-called “gut–liver axis” is used to describe the relationship between gut microbiota and the host liver [16]. It is unclear whether and how PFOS and its novel alternatives disrupt liver lipid homeostasis by altering the gut microbiota.

In this study, the effects of PFOS exposure on intestinal microbiota composition, lipid metabolism, and the gut–liver axis were systematically investigated in mice. This study not only deepens the understanding of the toxic effects of PFOS, but also provides important theoretical support for the prevention and treatment of metabolic diseases and inflammatory diseases caused by environmental pollutants.

## 2. Results and Discussion

### 2.1. Response of Intestinal Flora to PFOS Exposure

#### 2.1.1. Alpha Diversity Analysis

**Figure 1:**
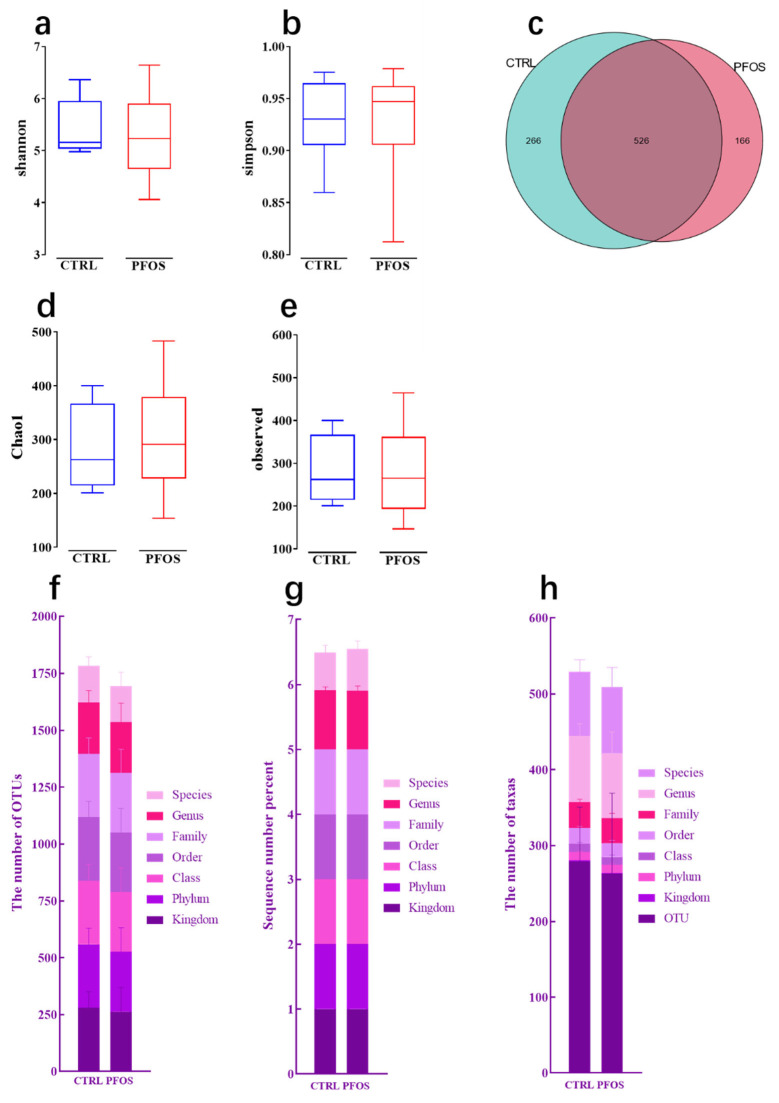
Alpha diversity and species metrics. (**a**) Shannon index. (**b**) Simpson index. (**c**) Shared and unique OTUs across the groups displayed by Venn diagram. (**d**) Chao1 index. (**e**) Observed features. (**f**) The number of assigned OTUs. (**g**) The sequence number percentage. (**h**) The number of taxa.

As can be seen from Figure 1, the species diversity of the PFOS group and CTRL group was compared by analyzing the α diversity of intestinal flora (including the Shannon index, Simpson index, Chao1 index, and number of observed features) (Figure 1a,b,d,e). The results showed that compared with the CTRL group, there was no significant difference in the species diversity of intestinal bacteria in the PFOS group (*p* > 0.05). The results showed that PFOS exposure had no significant effect on intestinal flora species diversity [17]. This suggested that PFOS did not exert significant selective pressure on the overall species composition of the gut microbiota, and could not cause significant changes in diversity. According to the distribution of OTUs (Figure 1c), there were 526 OTUs in the PFOS group and the CTRL group, 266 OTUs were unique to the CTRL group, and 166 OTUs were unique to the PFOS group, indicating that PFOS may lead to a decrease in the richness of some specific flora [18]. At different classification levels (Figure 1f–h), the total number of OTUs and taxa in the PFOS group was lower than that in the CTRL group, but there was no indication of statistically significant differences between the two groups (*p* > 0.05). In conclusion, PFOS exposure did not have a significant impact on the diversity of intestinal microflora species [18].

#### 2.1.2. Composition at Various Taxonomic Levels

**Figure 2:**
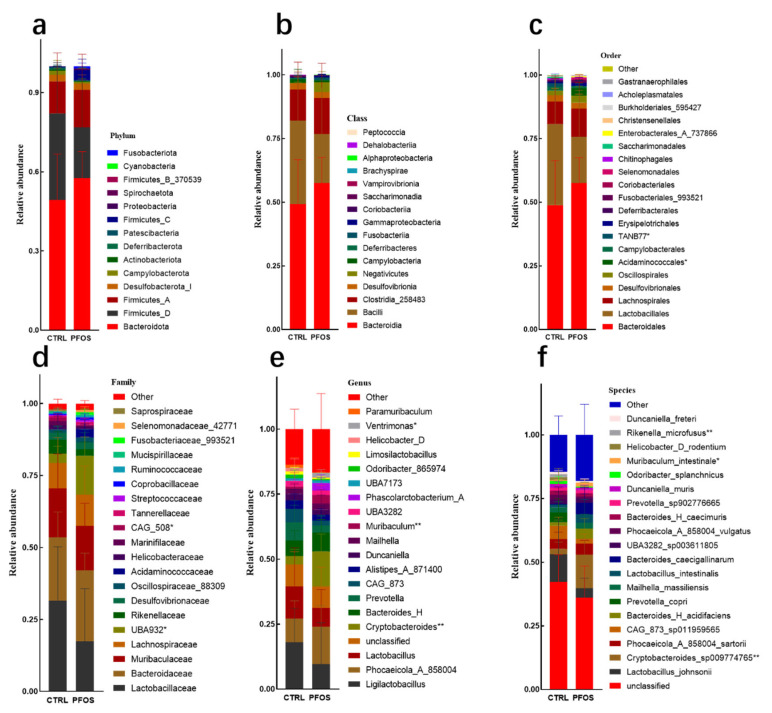
The different taxonomic grades of gut microbiota composition. (**a**) Phylum. (**b**) Class. (**c**) Order. (**d**) Family. (**e**) Genus. (**f**) Species. * *p* < 0.05. ** *p* < 0.01.

Since the main route of PFOS intake is through contaminated water and diet [19], which may affect gut microbiota, this experiment investigated whether PFOS exposure changes the composition of the microbiota in the gut. This study compared the composition of the gut microbiota at different taxonomic levels following dietary PFOS exposure. The results of Figure 2a–c show that the changes in Bacteroidota abundance had similar and consistent effects on the analysis results at the phyla, class, and order levels: the abundance increased. In the analysis of Bacteroidota at the phylum level, Bacteroidota, Firmicutes_D, and Firmicutes_A are the dominant phyla in the intestinal flora of mice, and the total relative abundance of these three phyla is over 90%. Compared with the CTRL mice, the level of Bacteroidota increased in the PFOS group, while Firmicutes_D and Firmicutes_A decreased (Figure 2a), but there was no statistical significance between the two groups (*p* > 0.05). PFOS may have a certain effect on intestinal flora structure, but the current data failed to provide statistical support. In the class-level analysis, after 21 days of PFOS treatment, Bacteroidales, Bacilli, and Clostridia_258483 were still the main components of the intestinal flora. Compared with CTRL group, the abundance of Bacteroidales in PFOS group increased, while that of Clostridia_258483 and Bacilli decreased (Figure 2b). In order-level analysis, Bacteroidales and Lactobacillales were the two orders with the highest relative abundance in both the CTRL group and the PFOS group. However, compared with the CTRL group, the abundance of Bacteroidales increased in the PFOS group, while Lactobacillales showed a downward trend. At the same time, in the PFOS group, the relative abundance of Acidaminococcales was significantly increased (*p* < 0.05), while the relative abundance of TANB77 was significantly decreased (*p* < 0.05) (Figure 2c). In the study at the family level, PFOS treatment increased the abundance of Ruminococcaceae and Bacteroidaceae families (Figure 2d), but there were non-significant differences between the CTRL group and PFOS group. The increased abundance of Ruminococcaceae has been suggested to characterize the progression of NAFLD and non-alcoholic steatohepatitis [20]. Bacteroidaceae increased with the severity of liver lesions [20]. Genus-level analysis (Figure 2e) showed a decrease in the abundance of the genus Ligilactobacillus, which is associated with lipid metabolism [21]. Under PFOS treatment, the abundance of Cryptobacteroides increased significantly (*p* < 0.01), while that of Prevotella decreased. In previous studies, Bacteroides and Prevotella acted as competitors, and there was an inverse relationship between their abundances in the PFOS group and CTRL group [22]. This suggests that PFOS treatment enhanced the competitive ability of Cryptobacteroides while inhibiting Prevotella, causing an imbalance of intestinal microbiota, which may affect the metabolism and health status of the host. At the species level (Figure 2f), in the PFOS group, the abundance of the Cryptooides_SP009774765 species was significantly increased (*p* < 0.01), while that of the Rikenella_microfusus species was significantly decreased (*p* < 0.01). In the previous related study, the abundance of Rikenellaceae increased significantly after exposure to PFOS [9]. This may be due to the fact that other species in the Rikenellaceae family may substantially increase under PFOS exposure, thus masking the decrease in the Rikenella_microfusus species. In addition, the abundance of the Muribaculum_intestinale species increased (*p* < 0.05). Thus, PFOS exposure alters the composition of the gut microbiota of mice, with effects across multiple taxonomic levels.

#### 2.1.3. Beta Diversity Analysis

**Figure 3:**
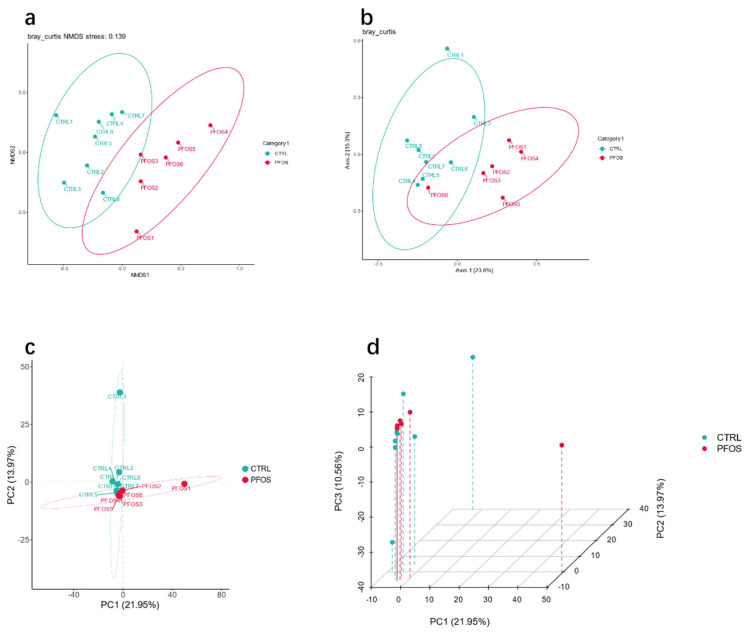
Gut microbiota beta diversity analysis. (**a**) Non-metric multidimensional scaling (NMDS). (**b**) Principal coordinate analysis (PCoA). (**c**,**d**) Principal component analysis (PCA).

In the study of gut microbial communities, the results of NMDS (Figure 3a) can directly demonstrate changes in community structure [23]. A clear separation between samples from the CTRL and PFOS groups was shown in the NMDS plot, suggesting that PFOS exposure significantly altered the community composition of the gut microbiome in mice. PCoA based on the unweighted UniFrac distance (taking into account the abundance of OTU) (Figure 3b) allows us to explore and visualize differences in microbial communities between the two groups [24], showing that the microbiome differences between the CTRL group and the PFOS group were larger than the intra-group differences, indicating significant differences in microbiome characteristics between the two groups. In addition, PCA, a commonly used linear dimensionality reduction technique, was applied to assess differences in ancestral frequencies between the PFOS and CTRL groups (Figure 3c,d) [25]. In the PCA 2-D and 3-D plots, in general, the samples from the CTRL group and PFOS group were clearly separated, indicating that PFOS exposure caused distinct changes in the intestinal microflora structure of mice.

#### 2.1.4. Analysis and Functional Statistics of RDA

**Figure 4:**
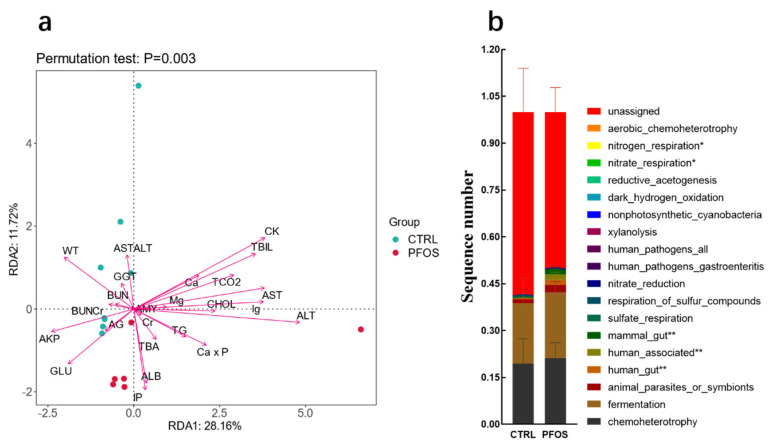
(**a**) RDA/CCA analysis. (**b**) KEGG database path prediction. * *p* < 0.05. ** *p* < 0.01.

Environmental parameters for each microbiome explain a different proportion of variation [26]. According to the results of the figure (Figure 4a), the explanation rate of variation accumulated by the two ranking axes is 39.88% (28.16% + 11.72%). This indicates that environmental factors have a certain influence on microbial community structure. The results of *p*-value (*p* < 0.05) further indicated that the relationship between environmental factors and microbial community was highly statistically significant, so the impact of environmental factors on community structure was indeed present and important. The graph clearly shows that CK, ALT, ALB, IP, GLU, AKP, and AST are significantly correlated with species distribution and community distribution. These environmental factors may reflect the metabolic changes in the host or pathological states such as abnormal liver function, which in turn affect the living environment of microorganisms. These results indicate that PFOS exposure may indirectly affect the population distribution and community structure of intestinal microorganisms by changing physiological indices in mice. In the analysis of KEGG database pathway prediction results, Figure 4b shows the functional differences between the CTRL group and PFOS group; in chemoheterotrophy and fermentation, PFOS exhibited higher levels compared to the CTRL group. In general, pathways to a PFOS-rich diet are associated with various fermentation and oxidation reactions and may reflect the oxidative stress level of the compound [17]. PFOS exposure enhanced chemical heterotrophic and fermentation functions in mice, not only reflecting the interference of PFOS on intestinal microbial metabolism and function, but also suggesting that PFOS may pose potential risks to host health through oxidative stress mechanisms. This provides a new perspective for further exploring the toxicity mechanism of PFOS at the microbiome and metabolic levels.

### 2.2. PFOS Causes Dyslipidemia in Mice

**Figure 5:**
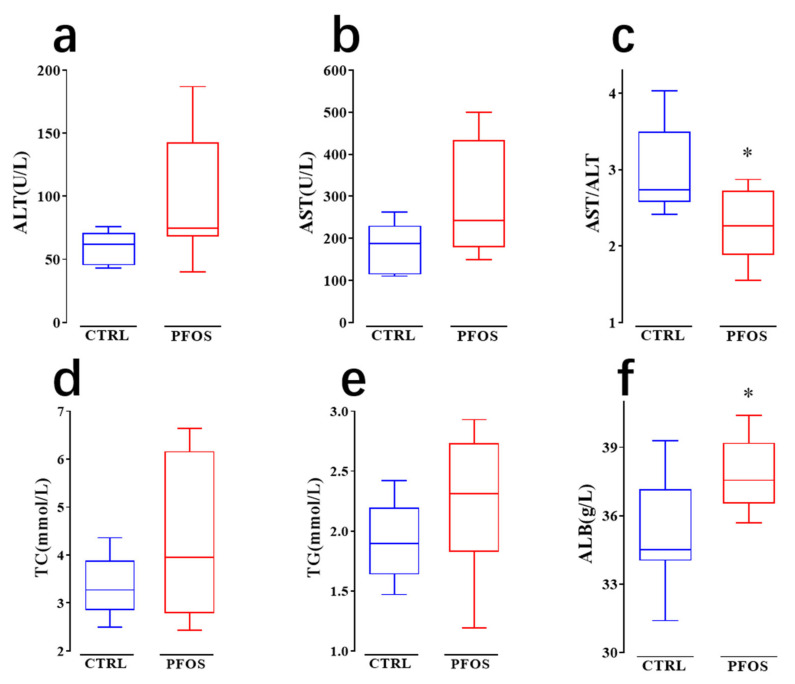
Effect of PFOS exposure on blood biochemical indices in mice. (**a**) Alanine aminotransferase (ALT). (**b**) Aspartate aminotransferase (AST). (**c**) Aspartate transaminase/alanine transaminase (AST/ALT). (**d**) Total cholesterol (TC). (**e**) Triglycerides (TG). (**f**) Albumin (ALB). * *p* < 0.05.

As can be seen from Figure 5a,b, the average levels of ALT and AST in the PFOS group were higher than those in the CTRL group, which is consistent with previous studies: there is a positive correlation between all PFAS and liver enzymes (such as ALT and AST) [27]. Serum ALT and AST levels are considered to be important indicators for judging the degree of dyslipidemia. Previous studies have shown that dyslipidemia is associated with elevated plasma ALT and AST levels [28]. Therefore, the increase in ALT and AST levels indicates that PFOS exposure can cause dyslipidemia in mice. This study also found that the AST/ALT ratio was significantly decreased (*p* < 0.05) (Figure 5c). A previous study in Japan showed that a low aspartate AST/ALT ratio was associated with increased cardiovascular disease and its risk factors [29]. This suggests that PFOS exposure leads to a significant decrease in the AST/ALT ratio, suggesting the potential for dyslipidemia and an increased risk of cardiovascular disease. In several epidemiological studies, elevated serum levels of PFASs (PFOS, PFOA, and PFNA) have been repeatedly associated with lipid disorders, mainly manifested by elevated blood cholesterol and TG [30,31,32]. As can be seen from Figure 5d,e, the average levels of TC and TG in the PFOS group were higher than those in the CTRL group, which are not statistically significant. This study also found that the level of ALB in the PFOS group was significantly higher than that in the CTRL group (Figure 5f), reaching a statistically significant difference (*p* < 0.05). One study showed that PFOS can strongly bind ALB [33]. PFOS bound to ALB may be transported to organs associated with lipid regulation (such as the liver and adipose tissue), thereby further affecting lipid metabolism and leading to changes in the lipid profile. For example, the strong positive correlation between PFOS and lipid profile may be due in part to this transport mechanism [34].

### 2.3. PFOS Causes Steatosis and Inflammation in Mouse Hepatocytes

**Figure 6:**
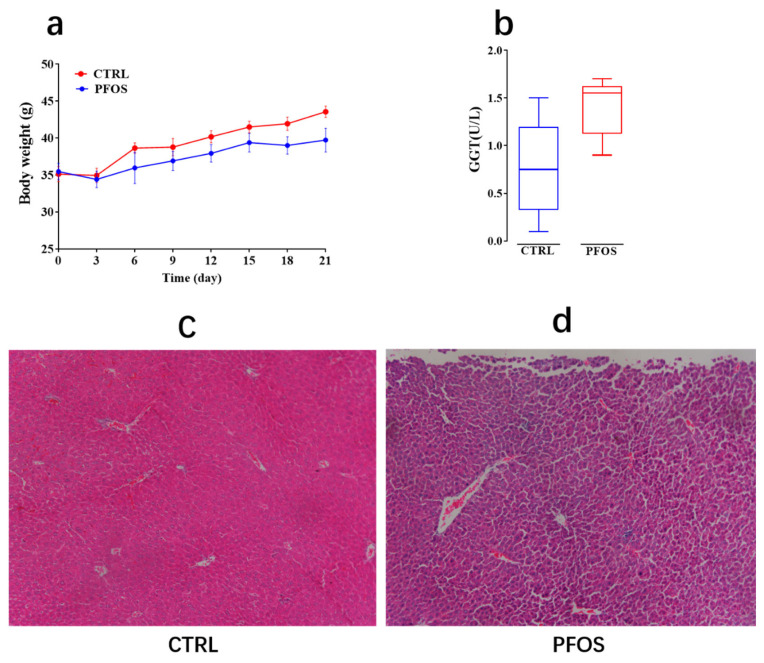
(**a**) Body weight. (**b**) Gamma-glutamyltransferase (GGT). (**c**) CTRL mouse liver representative histological section. (**d**) Representative histological sections of the liver of PFOS mice.

At the beginning of the experiment, weight was recorded every 3 days (Figure 6a). Results showed that PFOS exposure could reduce the weight of mice and had no significant effect on the weight change of mice (*p* > 0.05). This matches previous studies in which PFOS administration reduced body weight in mice fed a standard diet (SD) [35]. Comparing representative histological sections of mouse livers in Figure 6c,d, this study found that hepatocytes were tightly packed in the CTRL group, and no hepatocyte degeneration or inflammatory response was observed. However, after PFOS treatment, there was inflammatory cell infiltration, a small number of vacuoles in hepatocytes, and the arrangement of hepatocytes was disordered. These results indicate that PFOS exposure can cause steatosis and inflammation of hepatocytes in mice. Similar results were observed in previous studies [36]. It was analyzed above that PFOS exposure would cause dyslipidemia and increase TG level in mice. Previous studies have shown that PFOS can induce lipid accumulation in mice [15], and the disorder of lipid metabolism leads to excess TG accumulation in hepatocytes, thus inducing hepatic steatosis. PFOS can cause oxidative damage in the liver by inducing ROS formation and depletion of antioxidant defenses [37]. ROS is an important pathogenic factor in the pathogenesis of many liver diseases and is usually a byproduct of liver cell metabolism and detoxification. GGT primarily catalyzes the transfer of gamma-glutamyl groups from peptides to other amino acids, and steatosis with elevated plasma GGT is also seen in non-alcoholic steatohepatitis and NAFLD [38]. Figure 6b shows that GGT levels are upregulated after PFOS exposure, and GGT has long been known as an antioxidant enzyme and is a clinical biomarker of liver disease, indicating that PFOS exposure triggers oxidative damage of hepatocytes and promotes inflammation by inducing oxidative stress.

### 2.4. Effect of PFOS Exposure on Intestinal Lesions

**Figure 7:**
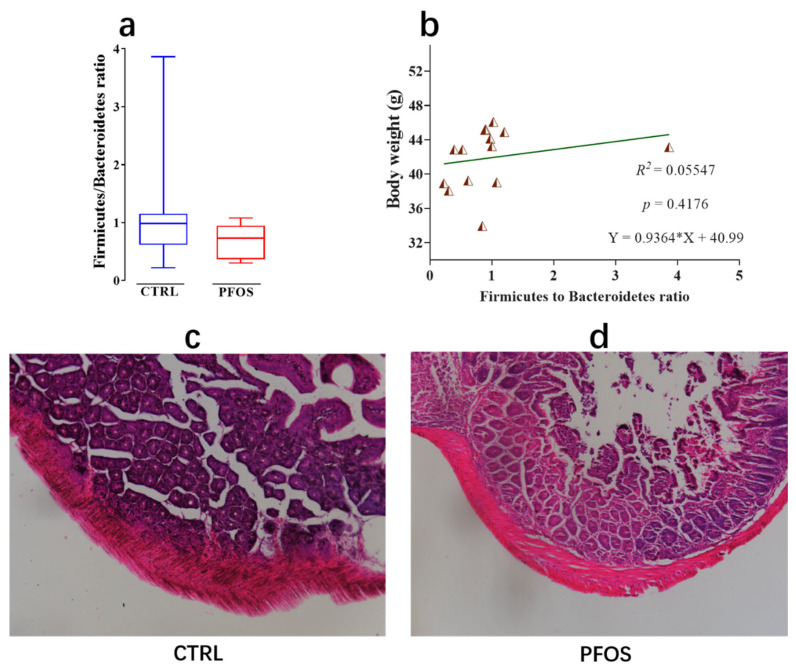
(**a**) Firmicutes/Bacteroidetes ratio. (**b**) Linear relationship between body weight and Firmicutes/Bacteroidetes ratio. (**c**) Representative histological section of the colon of CTRL mice. (**d**) Representative histological section of the colon of PFOS mice.

In this study, the Firmicutes/Bacteroides ratio was calculated, and a reduced Firmicutes/Bacteroides ratio was observed in PFOS mice, but there was no statistically significant difference compared to the CTRL mice (Figure 7a). Previous studies have shown that when correlations between body weight and gut microbial ecology were analyzed, a decrease in the Firmicutes/Bacteroides ratio was directly associated with weight loss [39]. In the above classification analysis of Bacteroidota, it was found in this study that the level of Bacteroidota increased in the PFOS group, while that of Firmicutes_D and Firmicutes_A decreased. Therefore, the Firmicutes/Bacteroides ratio was reduced. A reduced Firmicutes/Bacteroides ratio is associated with the development of IBD [40]. Bacteroidetes exhibit pro-inflammatory properties due to endotoxins and affect the production of cytokines that lead to IBD. In addition, Firmicutes bacteria have anti-inflammatory effects that can mitigate the progression of IBD. The linear relationship between body weight and the Firmicutes/Bacteroides ratio was also calculated in this study, as shown in Figure 7b: the relationship between body weight and the Firmicutes/Bacteroides ratio (R^2^ = 0.05547, *p* = 0.4176). This indicated that there was no significant linear relationship between the body weight and Firmicutes/Bacteroides ratio of mice exposed to PFOS in this study. Figure 7c,d show that PFOS mice developed histological lesions in their colons compared to CTRL mice. In the CTRL group, the colonic mucosal epithelial cells remained intact without significant swelling, the crypt structure was regular, the intestinal villi were intact, and the number of goblet cells was higher (Figure 7c). In contrast, PFOS group mice exhibited colon erosion, the glandular structure was destroyed, villus height and crypt depth were reduced, and goblet cells basically disappeared, accompanied by a large number of infiltrating inflammatory cells (Figure 7d). PFOS exposure led to colon formation in mice with lesions that were likely IBD. PFOS exposure induced pro-inflammatory infiltration-related IBD lesions [41].

### 2.5. Intestinal Flora Is Associated with Health Indicators

**Figure 8:**
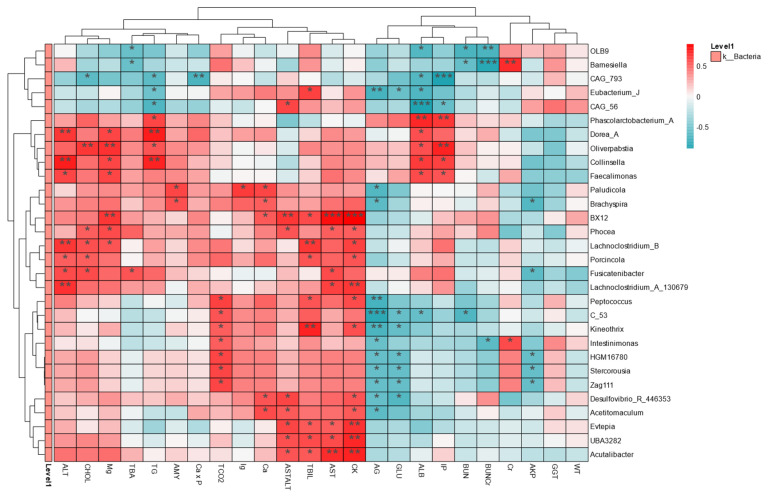
The relationship between gut microbiota (at the genus level) and phenotypes. R-values (rank correlation) and *p*-values are obtained by calculation. R-values are shown in different colors in the figure, and if the *p*-value is less than 0.05, it is marked with *. The legend on the right is the color interval of different R-values. Meanwhile, the color bar on the left indicates the phylum classification to which the species belongs. * *p* < 0.05, ** *p* < 0.01, *** *p* < 0.001.

To assess the relationship between phenotypes and specific gut microbiota, a heat map at the genus level was created in this study (Figure 8). A total of 30 gut microbiota were significantly associated with 25 health measures after PFOS exposure. BX12 and Phocea were significantly positively correlated with AST/ALT and AST, CAG_793, Eubacterium_J, and CAG_56 were significantly negatively correlated with TG and ALB, and Dorea_A and Collinsella were significantly positively correlated with ALT and TG. According to genus-level correlation heat map analysis, there was a significant correlation between intestinal microbiota and the selected health indicators. Interestingly, more species were closely related to ALT, AST, ALB, and TG, while fewer species were closely related to BUN, GGT, and BW (body weight). Therefore, PFOS exposure significantly affected the association between intestinal microbiota and health indicators, especially bacteria genera related to liver function (ALT, AST, ALB) and lipid metabolism (TG). This suggests that intestinal microbiota imbalance can disrupt the gut–liver axis, leadingto increased inflammation and impaired intestinal barrier [42]. Gut microbiota is a predisposing factor for the development and progression of NAFLD and IBD [43]. Therefore, the study of PFOS contributes to a deeper understanding of the impact of environmental pollution on human health, especially through the microbiome-mediated mechanism, and can better prevent and deal with metabolic and inflammatory diseases caused by environmental pollution and promote sustainable public health protection policies.

## 3. Materials and Methods

### 3.1. Animal Experiments

The animal experiments in this study strictly followed the ethical guidelines formulated by the Experimental Animal Ethics Committee of Quanzhou Normal University (QZTC20240518) to ensure the scientific and ethical design of the experiments. At the same time, the experiment process referred to the National Institutes of Health (NIH) “Guidelines for the Care and Use of Laboratory Animals” (NIH Publication No. 8023, revised in 1978) and the relevant regulations in the ARRIVE Guide.

The effects of PFOS on the gut microbiota and physiological health of 3-week-old male Kunming mice (with an average weight of about 20 g, provided by SLAC Laboratory Animal Center, Shanghai, China) were studied. The mice were randomly divided into CTRL group and PFOS treatment group, with a maximum of 8 mice in each group. The randomization of the mice was performed using a random number table method, and it was ensured that the experimental operator remained blind to the grouping information. The feeding conditions were strictly controlled with a humidity of 55% ± 5% and a temperature of 22 °C ± 2 °C, alternating between 12 h of light and 12 h of darkness. During the experiment, mice in the PFOS group were given 16 μg PFOS by intragastoria every day for 3 weeks. After the experiment, the mice were killed by cervical dislocation, and intestinal contents, blood, and liver tissues were collected for subsequent analysis.

### 3.2. Sample Collection and Processing

At the end of the experiment, the mice were weighed and sacrificed after overnight fasting, and blood samples were collected by ophthalmoscopy and centrifuged. The mice were then dissected, and liver and other important tissues were collected for morphological fixation, biochemical analysis, and histological detection. Part of the tissue samples were fixed in 4% paraformaldehyde solution, and the other part was frozen in liquid nitrogen for rapid preservation. Colon contents were collected under sterile conditions and frozen in liquid nitrogen for subsequent analysis.

### 3.3. DNA Extraction and PCR Amplification

Total genomic DNA was extracted from fecal samples by a CTAB-based method [44]. The extracted DNA was examined by agar-gel electrophoresis and adjusted to 1 ng/μL, followed by PCR amplification using specific primers targeting the V3–V4 region (341F and 806R primers) of the 16S rRNA gene. The amplified products were purified and verified for quality by electrophoresis, providing a high-quality template for subsequent analysis.

### 3.4. Generated Library and Sequencing

In order to comprehensively analyze the intestinal microbial community structure of mice, 16S rRNA gene sequencing was performed using the Illumina MiSeq PE250 platform (Illumina Inc., San Diego, CA, USA) in this study. Phusion^®^ High-Fidelity PCR Master Mix (Fermentas, Foster, CA, USA) was used for PCR amplification to ensure high precision and specificity. Processing of sequencing data was based on a standardized process using UPARSE software (Uparse version 7.0.1001) for de-noising, splicing, and redundant sequence culling of the original sequence. The 16S rRNA sequences were then divided into operational taxonomic units (OTUs) using QIIME or similar tools based on 97% sequence similarity criteria, which laid the data foundation for community diversity and structure analysis.

### 3.5. Analysis of Gene Expression

The gene expression analysis of liver lipid metabolism was performed by quantitative real-time reverse transcription polymerase chain reaction (qRT-PCR). Total RNA was extracted using an Omega RNA extraction kit to ensure high-quality RNA. The extracted total RNA was then reverse-transcribed into cDNA using the PrimeScript™ RT-PCR Kit produced by Takara, which was used as a template for subsequent qRT-PCR reactions to determine the expression level of the target gene.

### 3.6. Serum Biochemical Analysis

Serum biochemical analysis was performed using the high-precision Mindray Biochemical analyzer (Mindray BS-220, Shenzhen, China) and the commercial biochemical kit provided by Mindray Biomedical Electronics Ltd., (Shenzhen, China) covering a variety of key biochemical indicators including alanine aminotransferase (ALT), aspartate aminotransferase (AST), total cholesterol, triglyceride, albumin (ALB), and gamma-glutamyltransferase (GGT). In addition, liver lipid metabolity-related tests were performed using Abcam’s Ab65336 and Ab65390 kits for quantitative detection of total cholesterol and triglycerides in the liver.

### 3.7. Correlation Analysis

The correlation analysis of experimental data was completed by the Spearman correlation test, and the monotone relationship between variables and their statistical significance were evaluated by the rho value and its corresponding *p*-value. In order to further verify the correlation results, this experiment referred to previous correlation analysis studies [45], and used linear regression analysis to quantitatively analyze the relationship between microbiota changes and metabolic markers, and evaluated the intensity and direction of the influence through the regression coefficient and its confidence interval.

### 3.8. Histology

Liver tissue samples were fixed in 10% neutral buffered formalin solution and paraffin-embedded to prepare sections. Hematoxylin–eosin (HE) staining was performed to observe the morphological changes. Colon tissue was fixed in Bouin’s fluid and dissected continuously along the head and caudal axis. HE staining and hematoxylin restaining were used to further analyze the intestinal histological structure.

### 3.9. Statistical Analysis

In this study, non-metric multidimensional scaling (NMDS), principal coordinate analysis (PCoA), and principal component analysis (PCA) were used to analyze the differences among the samples. NMDS is based on the Bray–Curtis distance matrix, reduces the dimension by metaMDS, and selects the optimal dimension to minimize the stress value. PCoA adopts the Bray–Curtis or UniFrac distance, and uses pcoa to calculate and extract the eigenvalues of the principal axis. First, PCA normalized the data with Z-score, and then prcomp was used to calculate the principal component and contribution rate. All analyses were performed in R and Qiime2 and visualized via ggplot2. In addition, RDA/CCA analysis was used to evaluate the relationship between environmental factors and microbial communities, and functional prediction was performed using the Kyoto Encyclopedia of Genes and Genomes (KEGG) database. PICRUSt2 was used to predict the functional abundance of microbial communities and to analyze functional changes associated with metabolic pathways. Experimental data were expressed as mean ± standard deviation (Mean ± SD). The differences between groups were compared by the independent sample T-test, and when the *p*-value was lower than 0.05 (*p* < 0.05, *p* < 0.01, *p* < 0.001, respectively), the differences between groups were statistically significant. All statistical analyses were performed by GraphPad Prism 8 (GraphPad, La Jolla, CA, USA) or Wekemo Bioincloud (https://www.bioincloud.tech (accessed on 5 May 2024)).

## 4. Conclusions

This study demonstrates that PFOS exposure significantly alters the gut microbiota composition and affects host metabolism and inflammation through the gut–liver axis. While α diversity remained unchanged, β diversity analysis revealed a substantial shift in microbial community structure. PFOS exposure increased the abundance of Bacteroidota and decreased Firmicutes, with specific alterations in bacterial taxa linked to liver disease and lipid metabolism disorders.

Metabolic disorders caused by PFOS are manifested by elevated serum ALT, AST, and TG levels and decreased AST/ALT ratios, suggesting a heightened risk of dyslipidemia and liver dysfunction. Histopathological analysis confirmed hepatic steatosis, inflammation, and intestinal lesions consistent with IBD. Correlation analysis further highlighted strong associations between microbiota imbalances and metabolic dysfunctions.

These findings underscore the potential health risks posed by PFOS exposure, particularly in disrupting gut microbial homeostasis and promoting metabolic disorders. Future studies should explore long-term exposure effects and employ multi-omics approaches to further elucidate the mechanisms involved.

## Data Availability

All data generated or analyzed during this study are included in this published article.
